# Exploring the philosophical values of kimchi and kimjang culture

**DOI:** 10.1186/s42779-022-00136-5

**Published:** 2022-06-20

**Authors:** Reggie Surya, Anne Ga-Yeon Lee

**Affiliations:** 1 Food Technology Department, Faculty of Engineering, Bina Nusantara University, Jakarta, 11480 Indonesia; 2 Tourism Department, Université de Toulouse II-Jean Jaurès, 31058 Toulouse, France

**Keywords:** Kimchi, Kimjang, Food philosophy, Korea, Ethnic food

## Abstract

Kimchi is a traditional fermented vegetable dish from Korea globally appraised as healthy food. The most common kimchi is *baechu* kimchi made from Chinese cabbage (*Brassica rapa*). Having been an integral part in the Korean food culture for thousands of years, kimchi is considered as a symbol of Korean identity and pride. The importance of kimchi in Korean food culture is reflected from a special annual event dedicated to the making of kimchi held in autumn known as kimjang. It is a festive communal traditional practice of preparing large quantities of kimchi to be consumed throughout winter. Such an activity has been listed as UNESCO’s Intangible Cultural Heritage of Humanity since 2013. Indeed, the unique culture of kimchi and kimjang stems from the ancient wisdoms and old traditions of Korea that are rich in philosophy. This review discusses different philosophical values of kimchi and the kimjang culture in Korean traditions, including their accordance with the ancient philosophy of yin and yang, the five elements (wood, fire, earth, metal, and water), medicinal food, beauty, communal activity, filial piety, and humanistic values. Understanding the philosophical values of kimchi and kimjang culture would make people see kimchi not only as a mere ethnic food, but also as a global cultural heritage that needs preserving for its continuity in the future.

## Introduction

Consuming ethnic fermented foods is essentially an integral element in Asian food culture, particularly in Korea. Kimchi is a traditional fermented dish from Korea that has gained popularity at global level as healthy food. Basically, kimchi is a generic term in Korean for a group of unique traditional lactic acid-fermented vegetables [1]. The most common kimchi found in Korean cuisine is *baechu* kimchi (> 70% of kimchi present in Korean market) made from Chinese cabbage or napa cabbage (*Brassica rapa*), a vegetable created from years of natural crossbreeding between southern China’s bok choy cabbage and northern China’s turnip. Korean white radish (*Raphanus raphanistrum*) is the second most commonly used kimchi vegetable after cabbage (20%) [2]. The fermentation of kimchi often involves the addition of seasonings and spices to enhance its flavor, including red chili powder (*gochugaru*), scallions, ginger, garlic, sugar, salt, fish sauce, and fermented seafood (*jeotgal*). Due to the fermentation process and the presence of seasonings, kimchi is characterized by its palatability as giving spicy, sour, sweet, and carbonated tastes with a strong acidic odor [3].

For Korean people, kimchi is not only regarded as food, but also as the symbol of the nation’s pride and identity. Kimchi has been an integral part in the Korean food culture for thousands of years. Kimchi is rich in ancient historical values that reflect the Korean way of life. Kimchi was born in Korea and has grown ever since through different Korean civilizations, from the ancient to the modern one, thus evolving in harmony with Korean culture. The kimchi that we know today has gone through many development processes in terms of its identity, from merely fermented cabbages in brine solution to a complex and distinguished dish with various additional ingredients that has become the icon of Korea in the eyes of the world [4].

The importance of kimchi in Korean food culture is obviously reflected from a special annual event dedicated to the making of kimchi called kimjang, a unique traditional practice of preparing large quantities of kimchi to be consumed throughout winter. Kimjang is a communal activity that usually involves many participants, and the labor-intensive task is shared from a small-scale family level to a large-scale community level. Popularly known as Korea’s winter kimchi party, kimjang is one of the main holidays in the country and is considered to be the third biggest after Chuseok (Korean Thanksgiving) and Seollal (Lunar New Year) [5]. Such an occasion, that signifies grand celebrations and family gatherings in Korea, has been registered on the UNESCO’s list of Intangible Cultural Heritage of Humanity since 2013 [6].

This review aims to explore different philosophical values of kimchi and kimjang culture in Korean traditions. In the modern world of today, many Koreans still live by sticking to the ancient wisdoms and traditions, including eating kimchi on daily basis and participating in kimjang. It is essential to analyze the philosophical values of kimchi and kimjang culture since they are the determinant keys that give uniqueness to Korean food culture. There is no same or similar culture like kimchi and kimjang found elsewhere outside Korea since these elements root deeply from ancient Korean philosophy. Therefore, understanding the philosophical values of kimchi and kimjang culture would make people see kimchi not only as an ethnic food from Korea, but in a further manner, also as a historical Korean delicacy that tells a story of health and humanity. The values of kimchi also need to be dispatched to the younger generation of Korea to keep the traditions alive and grow a sense of pride in their heart as Koreans.

## History and originality of kimchi

The Korean peninsula is geographically isolated from neighboring countries: It is surrounded by rocky ocean fronts on the west, south, and east, and by rugged mountains on the north, despite being physically attached to mainland China. Throughout its history, Korea has also always maintained independence from China. Such conditions have allowed the ancient Koreans to develop unique ethnicity, language, and culture that are different from China, including in terms of food culture [7]. Since 2001, kimchi has officially been registered at Codex Alimentarius and gained an international recognition as the representative traditional fermented food of Korea [8].

**Fig. 1:**
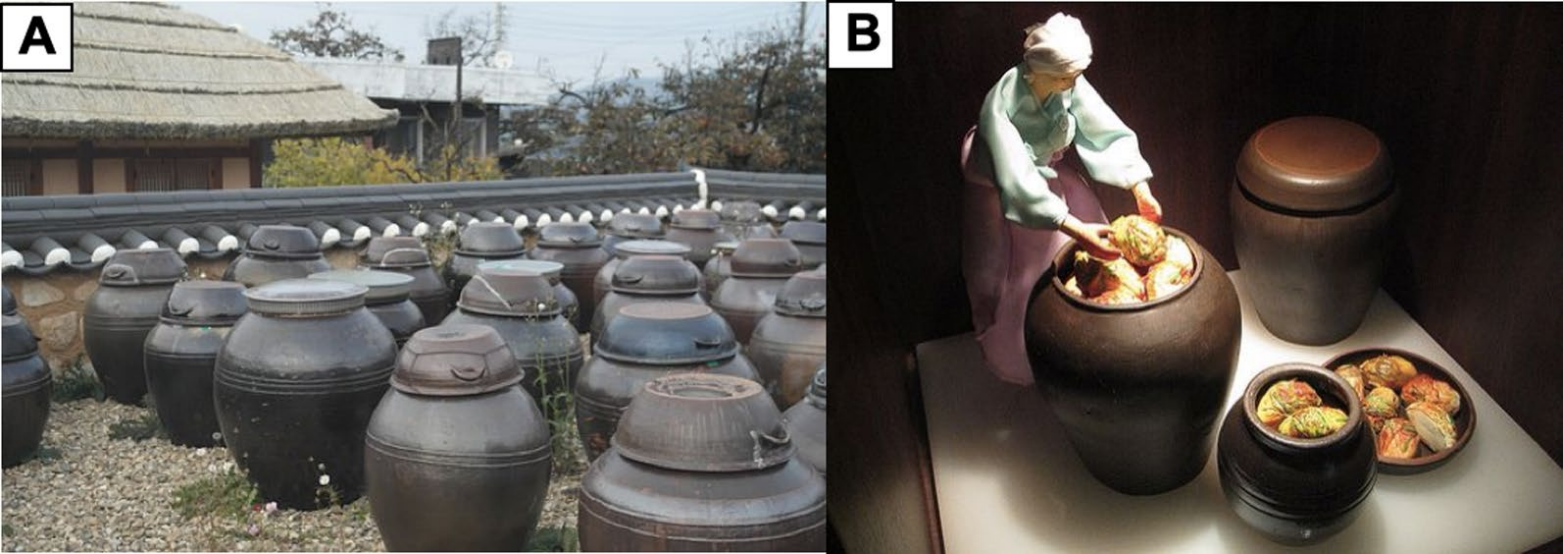
**A** Korean traditional earthenware jars known as *hangari* or *onggi* that are used to ferment and preserve vegetables, including to produce kimchi. **B** A diorama at Museum Kimchikan (formerly Kimchi Museum) in Seoul, South Korea, showing a Korean woman putting brined cabbages into earthenware jars to make kimchi

The food culture in Korea has been strongly connected to the nation’s long agricultural history that has lasted for more than 5000 years. The focus on agriculture has shaped the Korean diet to consist of mainly plant-based food [9]. The development of food processing technology in Korea was prompted by the urge to store and preserve food resources in order to protect crops from animals or birds and ensure food availability. Unlike in China where frying and pickling were the prevalent methods for food preservation, the limited production of cooking oils in Korea led ancient Koreans rather to opt for fermentation as a strategy for food preservation. Throughout years of experience, ancient Koreans discovered that some seasoned vegetables, fish, and salted beans remained edible and even developed a unique flavor after being kept in large earthenware jars called *hangari* or *onggi* (Fig. 1) [10].

Kimchi is the most important traditional fermented food in Korea. Historically, the tradition of making kimchi among Koreans started as a necessity of storing and preserving vegetables during the long harsh cold winters when many people died of starvation. Kimchi is suggested to be invented 4000 years ago according to the “*Sikyung*” (*Book of Odes*) published circa 500 BC. The character *jeo* (菹) appearing in such literature is believe to refer to kimchi. The oldest information about kimchi can be retrieved from ancient Korean literature entitled “*Samkuksagi*” (The Chronicles of the Three Kingdoms of Korea) published in 1145 AD stating that people already ate cabbage kimchi in the three states around 1500 years ago. Other ancient documents that highlight the existence of kimchi and its importance to Korean people include “*Naehun*,” “*Hunmongjahoe*,” “*Sinjeung-yuhap*,” and “*Kanicuckonbang*” [4, 11]. Therefore, until today, kimchi has been suggested to be present in Korean gastronomy for thousands of years [4, 12, 13].

Throughout its development, the existence of kimchi has been accompanied by false discursions challenging its originality [4, 12]. Firstly, kimchi was said to have the same root as the fermented vegetables generally known as *pao cai*. It is noteworthy that the characters in Korean language (*Hangeul*) are completely different from Chinese characters. Prior to the invention of *Hangeul* in 1433, Koreans used Chinese character in written documents while using Korean language when speaking. *Jeo* (菹), the Chinese character used by the Koreans to describe kimchi, is completely different from the one used to describe *pao cai* (泡菜). Considering the distinct characteristics between kimchi and *pao cai*, in 2021, a new Chinese character was invented to refer to the Korean kimchi, which is *xinqi* (辛奇) [14]. Secondly, kimchi was also said to be invented at the time of Imjin wars (Japanese invasion of Korea in 1592), during which red chili pepper was firstly introduced in Korea. Prior to such an event, kimchi was white and made without red chili pepper. This discursion was false because *gochu* (Korean red chili pepper), a special cultivar of *Capsicum annuum* commonly used to season cabbages in kimchi making, has been shown to exist in the Korean peninsula since 2000 years ago according to Korean official documents “*Samkuksagi*” and “*Mankiyoram*” [4, 12]. Both literature studies describe the *Chodo* as the island where *gochu* was planted. In addition, scientific evidence has demonstrated that *gochu* has existed in the Korean peninsula since billions of years ago and it is safe to say that *gochu* is original to Korea [12, 15]. Biologically, Korean *gochu* is different from the red chili peppers from Central American countries (such as Mexico and Colombia) and Southern Asian countries (such as India, Indonesia, and Thailand) that are too spicy to be applied in kimchi and *gochujang* (Korean spicy red chili pepper paste) [4, 12]. Korean *gochu* has a mild spiciness with a hint of sweetness and the Scoville heat unit (specific measurement unit for spiciness or heat of chili peppers) of < 1000 [4]. Finally, the last false discursion was articulated around kimchi being originally made of white radish and cabbage kimchi being developed only around 100 years ago. Such an assertion was not supported by scientific evidence since the “*Samkuksagi*” demonstrates the existence of cabbage kimchi in Korea 1500 years ago through a proverb “I want to tear a person limb from limb like the way we tear kimchi.” From such a description of tearing kimchi, one may conclude that the kimchi referred to in this document is cabbage kimchi [4]. Moreover, Chinese cabbage (*baechu*) has been cultivated in Korea for thousands of years [4].

## Gastronomical and nutritional aspects of kimchi

Gastronomy is defined as a cultural reflection based on norms, rules, beliefs, and values that is associated with the act of eating in a society [16]. In other words, gastronomy studies the relationship between food and culture. With its long history in the Korean culture and traditions, kimchi symbolizes the Korean food culture and is deeply rooted as part of Korean national identity [17]. In Korea, kimchi is indeed considered as a staple food besides rice. It is always present on Korean tables, and a Korean traditional meal is not complete without kimchi. Traditional Korean meals comprise a large variety of side dishes known as *banchan*, and kimchi is the most ubiquitous side dish consumed in two meals on a daily basis by a large proportion of Koreans [18]. Every day, a Korean is estimated to consume 27.6 g kimchi (25.0 g for males and 29.9 g for females) [19].

**Fig. 2:**
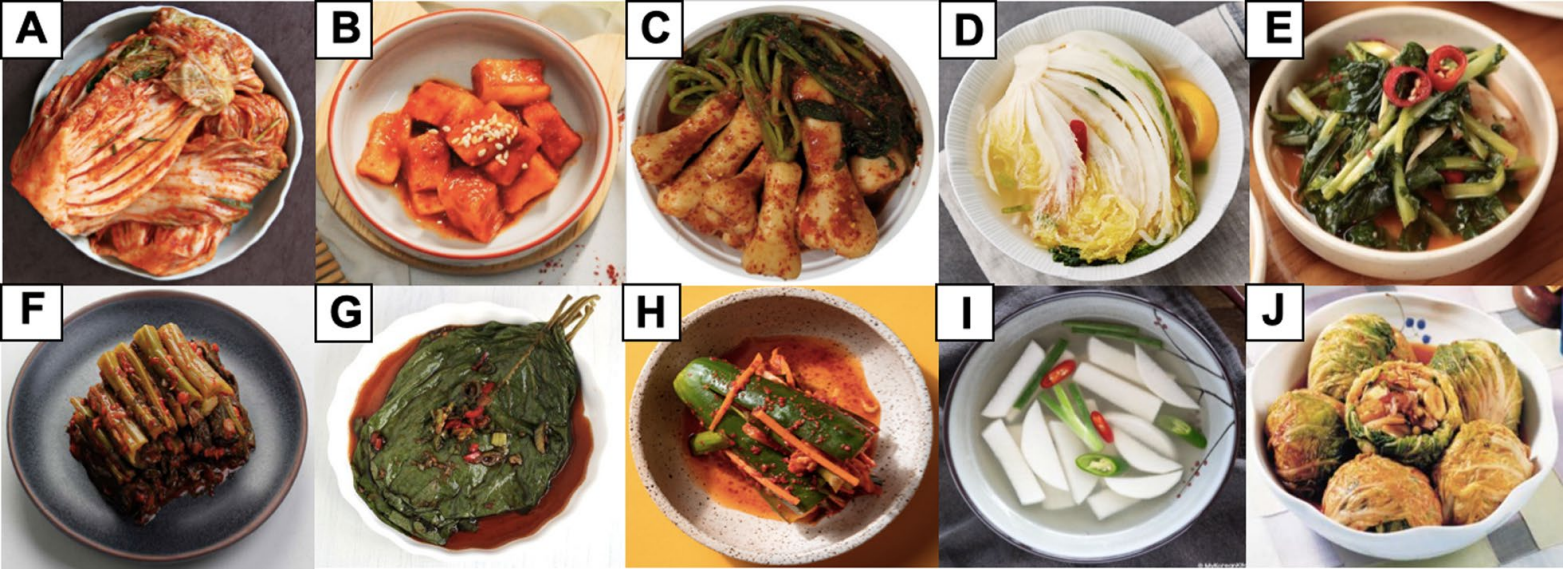
Some popular variants of kimchi: **A** *Baechu* kimchi, **B** *kkakdugi* kimchi, **C** *chonggak* kimchi, **D** *baek* kimchi, **E** *yeolmu* kimchi, **F** *gat* kimchi, **G** *kkaenip* kimchi, **H** *oi sobagi* kimchi, **I** *dongchimi* kimchi, and **J** *bossam* kimchi

It is suggested that there are about 200 types of kimchi in Korea that vary according to the different main ingredients used, regions where they were developed, and seasons [4]. Figure 2 recapitulates some major varieties of kimchi developed from different main ingredients. *Baechu* kimchi (Fig. 2A) made from Chinese cabbage is the most popular kimchi among Koreans and international consumers. It is often referred to as simply “kimchi.” Other popular types of kimchi are *kkakdugi* kimchi (Fig. 2B) made from diced Korean white radish and *chonggak* kimchi (Fig. 2C) made from ponytail radish [10]. *Tongbaechu* kimchi is *baechu* kimchi made from whole Chinese cabbages instead of the cut ones. *Baek* kimchi (Fig. 2D) is the version of *baechu* kimchi without red chili powder and, therefore, its color is mainly white. Young summer radishes are harvested when they are still soft and tender to make *yeolmu* kimchi (Fig. 2E). Other leafy vegetables can also be developed into kimchi, such as green cabbages into *yangbaechu* kimchi, green onion into *pa* kimchi, mustard leaves into *gat* kimchi (Fig. 2F), garlic chives into *buchu* kimchi, and perilla leaves into *kkaenip* kimchi (Fig. 2G). Green pepper and cucumber are cut and stuffed with chive and carrot to produce *gochu sobagi* kimchi and *oi sobagi* kimchi (Fig. 2H), respectively. *Dongchimi* kimchi (Fig. 2I) and *nabak* kimchi are two most popular examples of *mul* (watery) kimchi prepared with added water or brine solution through fermentation. Both are usually consumed as soup. *Bossam* kimchi (Fig. 2J), made from various ingredients considered as luxurious food items mixed with red chili pepper and salted yellow corvine, has a unique appearance since large cabbage leaves are used to wrap all the ingredients. A survey investigating the preference of kimchi types in Korean households revealed *baechu* kimchi as the most prepared and consumed type of kimchi, followed by *kkakdugi* kimchi, *dongchimi* kimchi, and then *chonggak* kimchi [20].

**Fig. 3:**
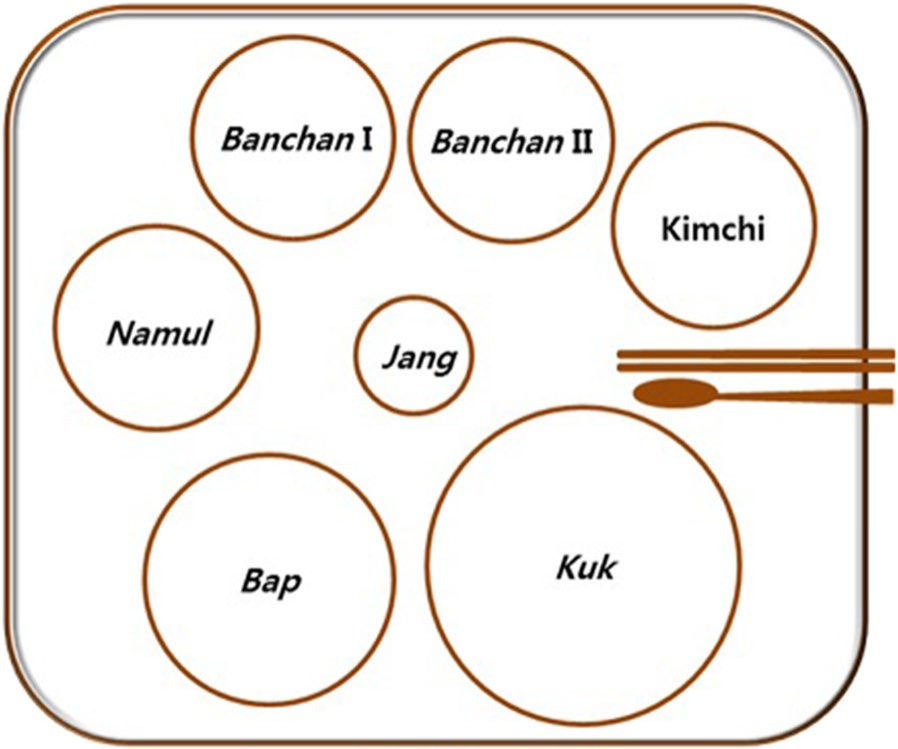
Illustration of a basic complete Korean traditional meal (bapsang) consisting of cooked rice (*bap*), soup (*kuk*), side dishes (*banchan*), greens (*namul*), condiments or salted dishes (*jang*), and kimchi [16]

In general, the basic traditional Korean meal (*bapsang*) consists of four constituents: cooked rice (*bap*), soup (*kuk*), side dish (*banchan*), and condiments or salted dishes (*jang*) [21] (Fig. 3). *Bap* provides carbohydrates as the main source of energy. *Kuk* is usually part of the main course, not an appetizer. It facilitates people to chew and swallow rice, thus supporting the digestive system. *Banchan* enhances the taste of the food and provides essential nutrition for the body. In the *bapsang*, *banchan* comprises usually one *namul* (greens), one type of kimchi, one vegetable dish (*banchan* I), and one high protein dish made from meat or fish (*banchan* II). *Jang*, such as fermented seafood (*jeotgal*) and chopped spices or herbs (*yangnyom*), is used to season food and stimulate the appetite. In general, individual settings of *bapsang* should be set up as follows: hot foods go on the right side and cold foods on the left. *Kuk* is placed to the right side and vegetables to the left. *Jang* is placed in front of the kimchi. Different from a western setting, forks and knives are not used on a Korean table. A long spoon (for *kuk*) and a pair of chopsticks made of metal (iron or stainless steel) are used instead as utensils.

As a traditional side dish, kimchi is usually served with other side dishes (*banchan*) in Korean family households and restaurants. Kimchi can be eaten alone or with rice, but it is also included in the recipes of other traditional dishes, including soup, porridge, and rice cake. Kimchi is also the basis for many derivative dishes, such as kimchi stew (*kimchi jjigae*), kimchi pancake (*kimchi buchimgae*), kimchi soup (*kimchi guk*), kimchi dumplings (*kimchi mandu*), and kimchi fried rice (*kimchi bokkeumbap*).

The production of kimchi consists mainly in fermenting vegetables (mostly cabbages or radishes) and other additional ingredients in a closed container preferably at low temperature to allow a slow microbial activity and long preservation. *Baechu* kimchi, the most popular type of kimchi, is made from cut Chinese cabbages that are coated with salt for 3–12 h to reduce the water activity essential for the growth of undesirable microorganisms. The excess water is then drained away, and seasonings are added to the brined cabbages. Some common seasoning ingredients used in the production of *baechu* kimchi include garlic, ginger, radish, carrot, scallion, and red chili powder (*gochugaru*). The presence of the latter brings uniqueness to kimchi and is the main differentiator of kimchi compared to other ethnic fermented vegetables originating from other countries, such as *pao cai*, *tsukemono*, *acar*, and *sauerkraut*. Finally, the seasoned brined cabbages are left fermented for 3–4 days at room temperature or 3–4 weeks at 4 °C in the refrigerator [11]. The main actors in the fermentation of kimchi are a broad group of bacteria know as lactic acid bacteria (LAB) that, as their name implies, produce lactic acid from cellulose in plant tissues. These bacteria include, but not limited to, *Bacillus mycoides*, *B. subtilis*, *Lactobacillus brevis*, *Lb. kimchii*, *Lb. plantarum*, *Lactococcus carnosum*, *Lc. lactis*, *Leuconostoc carnosum*, *Ln. citreum*, *Ln. kimchii*, *Ln. mesentroides*, *Serratia marcescens*, *Weissella cibaria*, *W. kimchii*, *W. koreensis*, and *W. soli*. Yeasts, such as *Saccharomyces* sp. and *Candida* sp., are also present in kimchi [22]. In the early fermentation stages, *Leuconostoc* sp. appears to be the most dominant due to its lower acid tolerance and microaerophilic properties. As the fermentation occurs, the rise in acidity favorizes the growth of bacteria with higher acid tolerance, mainly *Lactobacillus* sp. and *Weissella* sp. These microorganisms are present naturally at the surface of cabbages and other ingredients used. The step of salting as well as the addition of red chili powder (*gochugaru*) inhibits the growth of putrefactive and pathogenic bacteria, thus allowing the LAB to flourish and become the dominant microorganisms. The lactic acid produced by the LAB also lowers the pH to 4.0–4.2 and creates an acidic environment that cannot be tolerated by most other microorganisms that survived the salting process [23].

Kimchi has gained international appraisals as a healthy food. Kimchi has a balanced nutritional profile and is relatively low in calories [24, 25]. Regular kimchi consumption has been linked to the elevated health status and life expectancy of the Koreans [20]. Due to the variety of preparations possible for kimchi, its nutritional values may vary. The nutritional content of *baechu* kimchi is presented in Table 1 [26]. In general, kimchi is considered as an excellent source of fiber, vitamins, and minerals. Indeed, the vitamins and minerals in kimchi may vary depending on the vegetables used. Chinese cabbage contains a high amount of vitamin C and vitamin K as well as smaller amounts of iron, potassium, calcium, and copper. A kimchi recipe with carrots would contain vitamin A in a significant manner, while radishes would supply potassium, folate, and riboflavin [27]. Since kimchi is made with salt, one should be aware of its elevated sodium content. The carbohydrate content of kimchi would depend on the concentration of sugar added to it. Some kimchi recipes include the addition of sweeteners (such as honey or fruit juice) to balance out sourness. Kimchi is low in protein. However, recipes that include fermented seafood (*jeotgal*) would improve its protein profile. As kimchi is made from vegetables, it is naturally fat- and cholesterol-free.

**Table 1:** Nutritional values of cabbage kimchi (*baechu* kimchi) [26]

	Amount per 100 g	% Daily value
Energy (kcal)	33.9	2
Carbohydrates (g)	7.0	2
Sugars (g)	0.3	
Dietary fiber (g)	0.8	3
Protein (g)	1.1	2
Fat (g)	0.4	1
Vitamins		
Vitamin A (IU)	805	16
Vitamin C (mg)	4.4	7
Vitamin D (mg)	0.0	0
Vitamin E (mg)	0.5	2
Vitamin K (mcg)	7.5	9
Thiamin (mg)	0.0	3
Riboflavin (mg)	0.0	2
Niacin (mg)	0.6	3
Pyridoxine (mg)	0.1	5
Pantothenic acid (mg)	0.1	1
Folate (mcg)	29.5	7
Cyanocobalamin (mcg)	0.0	0
Minerals		
Calcium (mg)	22.2	2
Iron (mg)	0.7	4
Magnesium (mg)	12.4	3
Phosphorus (mg)	20.1	2
Potassium (mg)	84.2	2
Sodium (mg)	781	34
Zinc (mg)	0.2	1
Copper (mg)	0.1	3
Manganese (mg)	0.2	9
Selenium (mcg)	1.4	2

## Kimchi and kimjang culture: a taste of community spirit

**Fig. 4:**
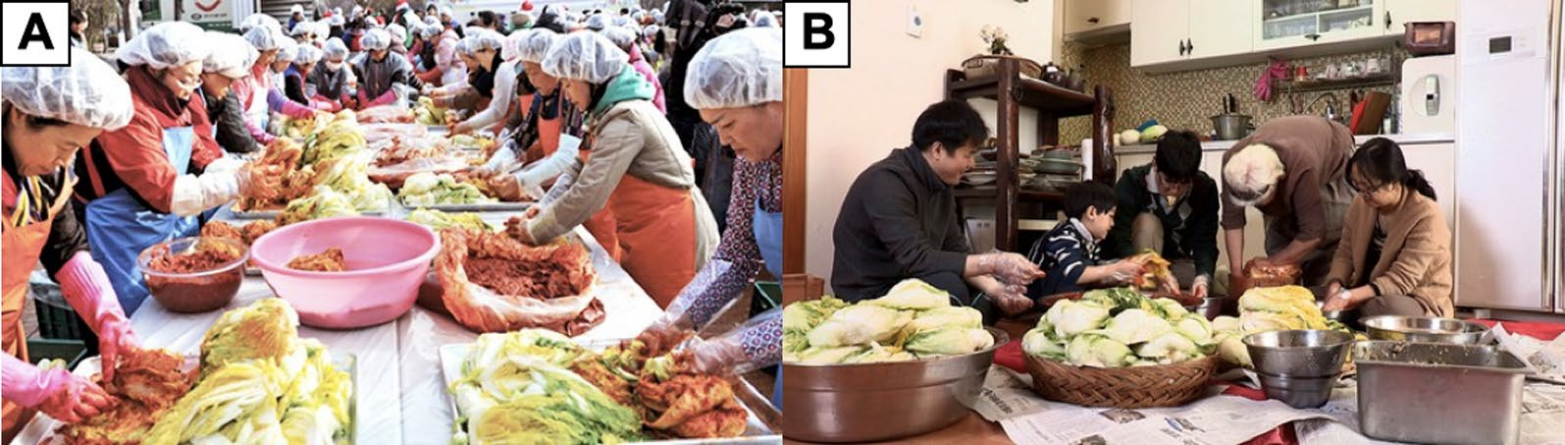
Kimjang, a traditional communal kimchi-making culture for consumption in winter that can be done at the community level (**A**) or family level (**B**)

Kimjang is a traditional Korean practice of preparing large quantities of kimchi to consume in the wintertime (Fig. 4). The tradition of kimjang stems back from thousands of years ago, when it was originally a family ritual in autumn to ensure the availability of kimchi during the long and harsh winter lasting for 3–4 months, when vegetables were scarce. The culture continued to somehow gain a broader audience, thus involving not only family members, but also the whole neighborhood, community, or village. The preparation of kimjang follows a yearly cycle. In spring, Korean households procure shrimp, anchovy, and other seafood for salting and fermenting. In summer, they buy sea salt for the brine. In late summer, red chili peppers are dried and ground into powder. Nearing winter, mostly in November and early December, various kimjang activities are organized in many places in Korea and gather a lot of people from different backgrounds, both males and females. Traditionally, male participants are expected to perform tasks that require strength (carrying large quantities of cabbages and radishes, digging holes to bury kimchi jars, and building a hut over jars), while female participants are mainly involved in the preparation of kimchi. Within a family, the whole operation of kimjang is usually under the control of the eldest woman of the family. Indeed, kimchi making and know-how constitute essential parts of the cultural tradition of a Korean household to be transmitted to next generations: from mothers to daughters and from mother-in-laws to daughter-in-laws. Through their participation in kimchi making, women are given cultural status in their community [5, 28, 29].

Kimjang is considered as a unique social experience since the tradition means more than a good deed of making and sharing kimchi with other people. Furthermore, it emphasizes the culture of sharing and the community spirit, two values embedded in the Korean tradition of kimchi making [30]. It is an activity where people’s personalities, emotions, and friendships are experienced in such a way that the event serves as a binding force for continuing and maintaining the relationships. Kimjang is a team work process of creating a common taste. All participants are involved in making seasonings, deciding the degree of saltiness of cabbages, adding ingredients to an agreed taste, and enjoying the final product together. By participating in kimjang, people build a cultural community that transcends regional and socioeconomic boundaries within society. Celebrated annually and passed down for numerous generations of families, kimjang allows Korean people to practice the spirit of sharing among while promoting solidarity and providing them a sense of identity and belonging [31, 32]. In 2013, kimjang was recognized as a world Intangible Cultural Heritage of Humanity by the UNESCO [6, 33].

**Fig. 5:**
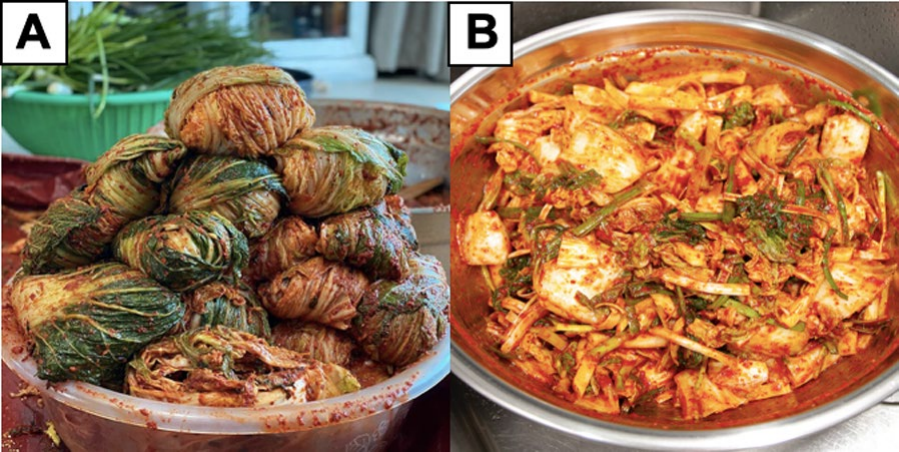
Different types of *baechu* kimchi based on the preparation manner of Chinese cabbages: **A** *pogi* kimchi made from whole cabbage heads whose leaves are neatly wrapped and stuffed and **B** *mak* kimchi made from small pieces of cut cabbages mixed with other ingredients

Kimjang is a laborious culinary activity that can span several days and requires the hard work of entire families or neighborhoods. The most common kimchi produced during kimjang is *tongbaechu* kimchi made from Chinese cabbage. During kimjang, cabbages are present in very large quantities. They are cut in half or quarter prior to soaking in salt water for a day and draining. A brightly red marinade is prepared using ginger, garlic, radish, carrot, green onions, starch, and red chili powder (*gochugaru*). Anchovy extract and fermented prawn paste can also be added for extra richness. It is noteworthy that in traditional kimjang, the chopping and mixing of all ingredients is done manually by hand, a painstakingly long affair with no shortage of hard work. Oftentimes, the whole process of kimchi making also involves squatting down for a prolonged period, thus resulting in muscle aches. After mixing all the ingredients, kimjang participants apply a thick layer of the marinade to every leaf of the cabbage head before wrapping the cabbages in a certain manner known as *pogi* (Fig. 5A). In the past, the wrapped cabbages were then stored in large quantities in large clay jars kept outside the houses (Fig. 1) or sometimes were buried underground and covered by straws. However, as many Koreans live in modern apartments today, kimchi is kept in small containers and often in designated kimchi refrigerators invented in the 1980s. Today, most Korean households possess at least a kimchi refrigerator. A typical average-size extended Korean family would prepare fifty or so heads of *baechu* kimchi during kimjang to be consumed gradually throughout the winter. Kimjang kimchi is considered a delicacy in Korea. Since a kitchen is often not spacious enough to accommodate all the participants, kimjang is mostly done in living rooms, gardens, backyards, and even rooftops. On a larger scale, kimjang requires a lot of people and space for all the paraphernalia (giant tubs, big buckets, huge sieves, and plenty of elbow room). Interestingly, kimjang does not end when the last head cabbage is wrapped and ready. After a full tiring day of kimchi preparation, a big feast usually follows with Korean traditional foods (usually steamed pork belly and fresh kimchi) and Korean rice wine (*makgeolli*) consumed amidst conversations, songs, and laughter. Furthermore, distributing boxes of kimjang kimchi to friends, coworkers, and relatives is common and harks back to the days when most Koreans lived in villages and shared their kimchi with almost everyone in the neighborhood as an act of generosity. Today, many kimjang activities are held for providing kimchi to the poor or the disadvantaged [30].

More than a communal food preparation, kimjang is a form of socializing where people come together as an entity to make large quantities of kimchi that will go around to everyone in the community for a whole year. The process is about achieving solidarity as a community. It is a beautiful moment of sharing, when people have small talks about joy, concerns, love, anger, and all the emotions that make up their life. It might be the moment where people feel more alive, energetic, and fun, reminding themselves once again the nature of humans as social beings. All the families, friends, and neighbors joining kimjang hope for a time well spent and a moment of no regret. This is a special moment yearned by many Koreans: to see family members, friends, or relatives living far away being intentionally present for the occasion. Cherishing the present and letting time pass by is what many Koreans still contemplate since the old times to this day. Kimjang flourishes with Koreans’ spirit and their ideas of aging, living wisely, and meeting the end in peace [34].

Nowadays, kimjang faces many challenges as a result of modernization that might threaten its continuity. Modern agriculture has allowed to grow cabbages and radishes all year independent on the seasons. Hence, kimchi is practically always available at the market all year long, including in winter. In addition, the reducing Korean population, modern apartment living, fewer family members, and convenience-oriented lifestyle have made modern Korean people reluctant to participate in kimjang. Despite all these factors, kimjang still survives and many Koreans still realize the cultural significance and social values attached to it in the Korean society. Kimjang still becomes a coveted moment and is still held in many parts of Korea. The COVID-19 pandemic in 2019–2021 has indeed been a bitter blow to many Koreans as a series of restrictions for social activities prevented them from gathering as they usually would do for kimjang [24]. Once the pandemic subsides, however, people would finally reunite for this joyous festival of kimchi, as well as the quintessential manifestation of the Korean traditional sense of community. It is predicted that kimjang will last in Korean culture despite the current modernization and will surely continue adjusting to social changes. Therefore, kimjang in the future might be different from what it is today [5].

## Philosophical values of kimchi and kimjang culture

As two connected elements that have been embedded in Korean culture for thousands of years, kimchi and kimjang culture was developed from the core philosophy of Korean people. The philosophical values of kimchi and kimjang discussed in this manuscript would explain how kimchi has been shaped through times into the regular kimchi we know today.

## Kimchi adopts the concepts of harmony and balance in the nature

Korean traditional food is close to nature, seasonally driven, and centered on vegetables, which compose the major part of Korean meal. At the beginning, fermenting vegetables in Korean food culture arose as a result of long and harsh winters impeding ancient Koreans from looking for foods in the nature for a long period. To survive, they developed fermentation as a preservation method for vegetables to ensure sufficient vegetable provision amidst the winter. Therefore, kimchi symbolizes the value of perseverance built on love and care for providing foods to the whole family in difficult winter times [35]. Indeed, the culture is still alive in the modern days with the existence of kimjang.

Kimchi is traditionally prepared from local fresh ingredients harvested just a moment before the preparation begins. Korean kimchi makers realize that the freshness of the ingredients is a key factor to produce a perfect kimchi. The best kimchi is born from high-quality freshest vegetables. Fresh vegetables still have a firm texture that supports the crunchiness of kimchi [36]. Moreover, fresh vegetables are alive and contain thousands of microorganisms and active enzymes that would induce favorable chemical reactions during the fermentation. Old or wilted vegetables have developed a soft texture and unpleasant flavor resulting from enzymatic and microbial activities [37]. The fermentation of kimchi is indeed a work of nature. It is called a spontaneous fermentation since no microbial culture is added to the ingredients on purpose. Therefore, the growth of lactic acid bacteria and other microorganisms during fermentation takes place according to the “nature’s intention.” Because the fermentation of kimchi relies on the activities of microorganisms naturally present in the raw materials, the initial microbial population on the surface of vegetables plays an important part in determining the success of the fermentation process. Vegetables that are not fresh are dominantly colonized by spoilage microorganisms and using them in making kimchi would lead to an unbalanced microbial profile leading to failure to make a good kimchi [38]. Philosophically, using fresh ingredients to make kimchi is also a form of respect and gratitude towards nature. We thank God and the nature for giving us blessings in the form of fresh vegetables by making out from them a valuable kimchi dish. We also respect the food by treating the vegetables with love and joy, giving them an aesthetic appearance, and allowing them to transform elegantly into kimchi with a greater value. Prepared using fresh seasonal vegetables locally grown in the Korean land and seas that are harvested according to nature’s timetable, kimchi is a healthy food nurtured by the sky, embraced by the earth, and made by people.

Since Korea has a temperate climate with four distinct seasons, different types of kimchi are made traditionally according to the availability of the seasonal vegetables. Although the existence of modern kimchi refrigerators has made this seasonality unnecessary nowadays, Korean people continue to consume kimchi according to traditional seasonal preferences [39]. This is done mainly due to the kimchi-eating custom that has passed from generations to generations. Traditionally, the greatest kimchi varieties appear during the winter. *Dongchimi* kimchi is largely served at the beginning of winter. In preparation for the long winter, many types of kimjang kimchi are prepared in early winter and stored at low temperature to ensure a slow fermentation process and prolong preservation. By doing this, kimjang kimchi can be eaten for months before being too sour and inedible, long enough to ensure the vegetable intake during the winter. In spring, after a long period of consuming kimjang kimchi during the winter, fresh leafy greens appear and are used to make kimchi. These kinds of kimchi are mostly consumed fresh (without fermentation) as salad. *Nabak* kimchi made from young radish is commonly eaten in spring, mostly with rice cake soup (*tteokguk*) to celebrate New Year. In summer, the abundance of *yeolmu* radish, leek, and cucumber promotes their use as main ingredients of kimchi. In autumn, the production of *baechu* kimchi starts and begins to increase since due to the growth of Chinese cabbages on Korean lands. Another popular type of kimchi widely consumed in autumn is *gogumasoon* kimchi made from sweet potato stems.

Unlike various seasonal kimchi, kimjang kimchi made in winter is made of vegetables from all four seasons. The main ingredients (cabbages and radishes) grow from late summer to autumn. Red chili pepper is a summer plant and Koreans take advantage of the summer heat and sunshine to produce dried red chili powder (*gochugaru*). Garlic is a winter aromatic plant in the previous autumn and has survived through winter to be harvested in summer. Spring is the season to procure shrimp, anchovy, and other seafood to be salted, fermented, and used as seasoning for kimchi. As such, traditional kimjang kimchi is often considered to be a wholesome fermented food containing different elements from all four seasons in it [39].

## Kimchi according to the yin and yang philosophy

**Fig. 6:**
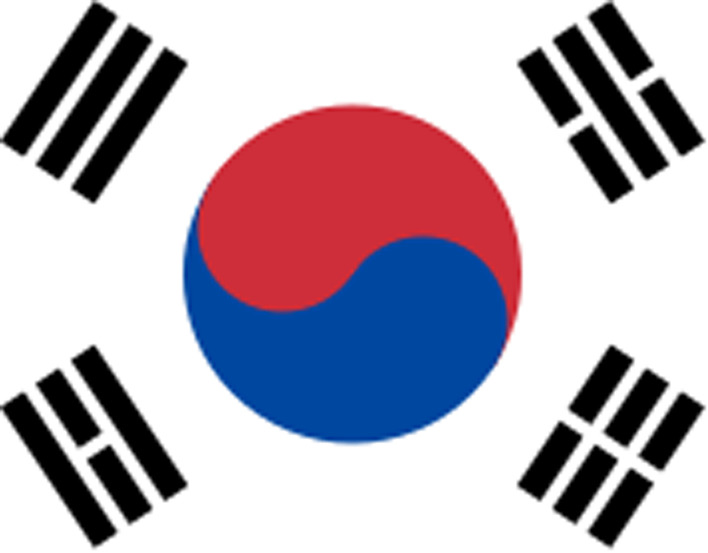
The flag of South Korea, also known as the *Taegeukgi*, consists of three main parts: a white rectangular background, a red and blue *taegeuk* in its center, and four black trigrams, one in each corner. The presence of the *taegeuk* symbolizes the importance of the yin and yang philosophy for Korean people

The yin and yang philosophy (or *eum* and yang philosophy in Korean) was born from Taoism and Confucianism, two of the major strands of Chinese and Korean philosophy and religion. It is an ancient complex relational concept that has developed over thousands of years. Briefly, the meaning of yin and yang is that the universe is governed by a cosmic duality, sets of two opposing and complementing elements in nature: the moon and the sun, female and male, cold and hot, dark and bright, passive and active, etc. [40, 41]. The philosophy of yin and yang is also obviously concretized in the prominent red and blue *taegeuk* in the middle of the South Korea’s national flag (Fig. 6).

The balance of yin and yang is important. If yin is stronger, yang will be weaker, and vice versa. Such a balance is perceived to exist in everything existing in nature, including food. As in food, the yin and yang concept classifies food into these two categories. Yin food is often associated with having a soothing effect while yang food is associated with having a warming effect. Both of them should be consumed proportionally, or else it may result in illnesses. In terms of flavor, yin and yang foods have their respective characteristics. In general, yin food has a bland, bitter, sour or salty flavor while yang food boasts a sweet or hot flavor. In every Korean meal, the presence of both yin and yang foods creates a unique combination of flavor that will not only pamper the taste buds but also contribute to health by balancing the corporal energies. To stay healthy, one should keep the balance between yin and yang in their body. If yin is too high in the body, yang food should be preferred to compensate it and vice versa [42].

Kimchi is a perfect example of a balanced food since its ingredients represent both yin and yang elements. The main ingredients of kimchi such as cabbages and radishes are naturally watery, cold, bland, and bitter, thus representing the yin foods. In contrast, the seasonings and spices, mainly the red chili powder (*gochugaru*), are considered as yang foods as they bring heat and spiciness [43]. These yin and yang ingredients are combined together in kimchi to create a unique sort of balance that embodies the health philosophy of yin and yang.

## Kimchi according to the philosophy of five elements

According to the Taoist philosophy, health is a state of balance in which yin-yang and five elements are at equilibrium. The concept of yin and yang underlies the philosophy of the five elements (wood, fire, earth, metal, and water) that shape everything in the universe in balance, including our health. These elements are then translated into different aspects, including the organs, emotions, colors, and tastes. With regard to color and taste, the wood element is green/sour, fire is red/bitter, earth is yellow/sweet, metal is white/spicy, and water is black/salty [44].

The concept of the balance among the five elements is traditionally applied in Korean cuisine through the food serving that implies foods of different tastes and colors combined together to make a unique meal (Table 2). In a basic meal, kimchi provides a complex and rich taste comprising the five taste elements together: sweet from the sugar, salty from the salt and sauce, sour from the acids, spicy from the red chili pepper, and bitter from the natural compounds present in cabbages and radishes. The five flavors are believed to be associated with specific organs of the body that are also interconnected through the yin and yang philosophy, thus are known as yin organs and yang organs. Sweet food is good for the stomach and pancreas, sour food is good for the liver and gallbladder, salty food is good for the kidneys and bladder, bitter food is good for the heart and small intestine, and spicy food is good for the lungs and large intestine [44]. If an organ is ill, the food of the same element would help repair the damages of the organ.

**Table 2:** Contribution of popular kimchi ingredients in terms of colors and tastes derived from the philosophy of the five elements

		Element/color				
		Wood	Fire	Water	Earth	Metal
		Green	Red	Black	Yellow	White
*Element/taste*						
Wood	Sour	Fermented cabbage				Fermented cabbage
Fire	Bitter	Scallion, watercress, crown daisy, mustard leaves				Cabbage, radish
Water	Salty		Fermented seafood (*jeotgal*)	Soybean sauce, fish sauce, pickled anchovy	Dried squid, yellow corvina	Salt, flour/starch (thickening agent), white sesame seeds
Earth	Sweet		Carrot, jujube	Black sesame seeds	Carrot, ginkgo nut	Cabbage, radish, sugar, pear, apple, pine nut
Metal	Spicy	Scallion or green onion	Red chili powder (*gochugaru*)			

**Fig. 7:**
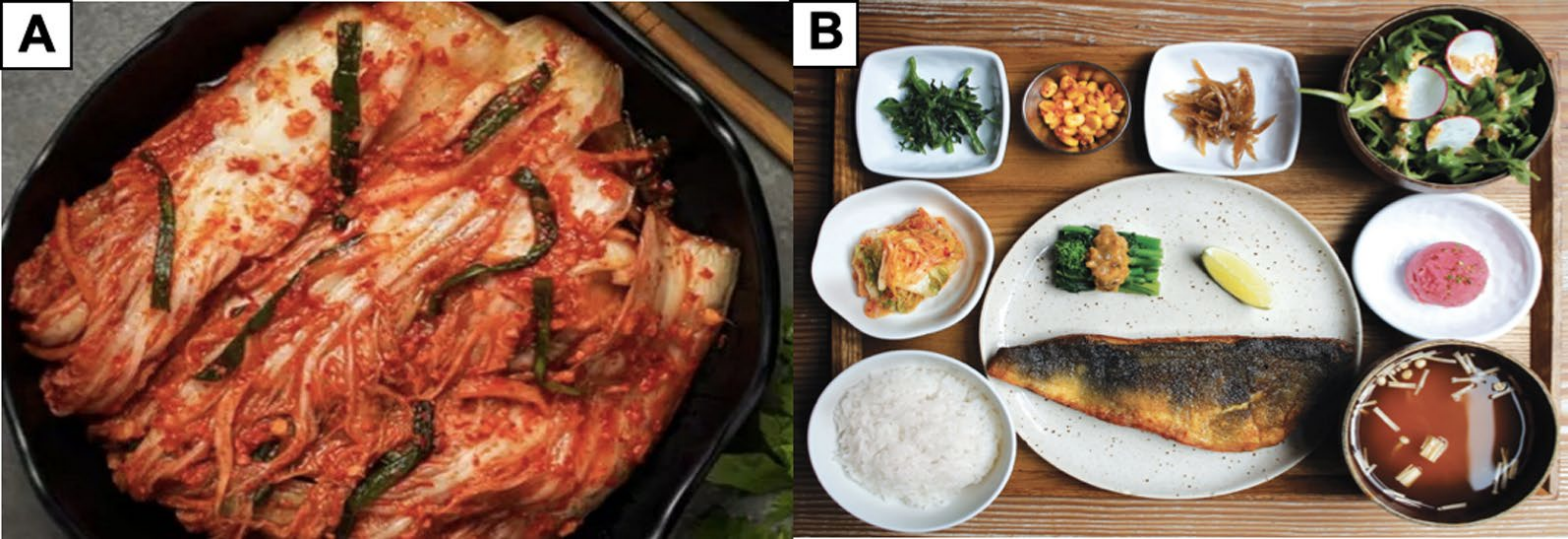
**A** Kimchi is made from ingredients of different colors that respect the philosophy of the five elements and **B** a complete Korean meal with kimchi presents the complete five different colors according to the philosophy of the five elements: red, white, green, yellow, and black

Kimchi also brings different colors of the five elements to a Korean meal, mainly red and white due to the presence of the predominantly white main ingredients (cabbages and radishes) and brightly red chili powder. In many cases, other colorful ingredients are also added to kimchi, including carrot (orange/yellow), scallions (green), and soy/fish sauce (black), thus balancing the five elements in kimchi preparation (Table 2 and Fig. 7A). A complete Korean meal is usually aesthetically colorful and comprises dishes and garnitures formed by the five colors of the five elements (Fig. 7B). Indeed, naturally colored foods, mostly vegetables and fruits, are rich in phytochemicals know as antioxidants that have been proven to be beneficial to human health, such as slowing down aging, strengthening immune system, reducing risks of cardiovascular diseases, and even preventing cancer. These antioxidants include flavonoids in cabbages and radishes, carotenoids in chili powder, and chlorophyll in green vegetables [45].

## Kimchi embraces beauty

Koreans consider carefully both the taste and visual appearance when preparing food, as reflected in an old Korean proverb saying “*bogi joeun tteogi meokgido jota*” that is translated into “what looks good tastes good” in English [46]. Kimchi exhibits a beautiful visual appeal through the addition of other ingredients bringing different colors as the embodiment of the five elements (Fig. 7A). *Tongbaechu* kimchi commonly made from whole Chinese cabbage during kimjang kimchi is aesthetically wrapped and arranged in a particular manner. The preparation technique is known as *pogi*, consisting in taking the whole quarters of brined cabbage and then stuff the individual leaves (Fig. 5A). This is much harder and more time consuming to do compared to *mak*, another preparation technique applied to *baechu* kimchi that implies cutting the cabbages into smaller pieces first before mixing them together with other ingredients [47] (Fig. 5B). *Bossam* (wrapped) kimchi (Fig. 2J) is a perfect example of an aesthetic kimchi. This dish, originating from Gaeseong in the Gyeonggi-do Province of Korea, is considered to be an elegant and luxurious delicacy since it is made from a plethora of delicious ingredients in addition to cabbages and chili powder, including seafood (baby octopus, oyster, and abalone), chestnuts, pine nuts, jujubes, watercress, green onions, mushrooms, gingers, apples, pears, and ginseng. To assemble a *bossam* kimchi, all the ingredients used for the filling are carefully arranged on a single leaf of local Gaeseong cabbage (that is broader than the Chinese cabbage’s) which is then wrapped into a big bundle and served in a bowl. *Bossam* kimchi is considered to be the most nutritious of all kimchi varieties and is usually consumed for special occasions and holidays [48].

Kimchi is also believed to exhibit the aesthetics of waiting slowly since its production involves a long fermentation process [46]. Through the fermentation process, the brined cabbages and radishes transform into kimchi. Its flavor becomes enriched with new flavors resulting from microbial activities and incorporation of other present ingredients. With the time, kimchi ages, develops more complex flavors, and gains its beauty as it becomes more precious. If a saying of “aging like fine wine” exists in the western culture, then “aging like a fine kimchi” might also apply.

## Kimchi is a medicinal food

A foundational philosophy of Korean cuisine is “*yak sik dong won*,” which literally means “food is medicine.” Throughout the ages, Koreans have been told about the importance of food for life, that food and medicine come from the same source. In other words, Korean people realize that the food they consume is intimately associated with their health. Thus, eating good food will bring them good health while eating bad food will bring them disease [44]. Indeed, Korean cuisine has a renowned global image as being healthy. Numerous scientific studies have suggested that the health benefits of Korean food were due to the diversity of natural ingredients, low overall calorie intake, and balanced nutrition compared to western food [10]. Korea is among the 10 countries with the highest average life expectancy in the world (> 80 years) [49], and such a phenomenon is associated with the high daily per capita consumption of greens (*namul*) in Korean cuisine [9].

Kimchi has been widely demonstrated in a scientific manner to exert many health benefits for humans. In addition to containing a balanced nutritional profile, kimchi is rich in lactic acid bacteria (LAB) generally known as probiotics, which are good beneficial live bacteria that are intended to provide health benefits when consumed [50]. Health functionality of kimchi includes anticancer, antiobesity, antiaging, colorectal health promotion, blood lipid reduction, immune promotion, brain health promotion, skin health promotion, and probiotic properties [2]. The only caveat to the renowned health benefits of kimchi would be its relatively high concentration of sodium due to the high amount of salt applied in the brining process. Indeed, the sodium intake among Koreans is very high (almost 4300 mg/day) [51] and exceeds the recommended limit of sodium daily intake of 2000 mg according to the World Health Organization (WHO) [52]. An average Korean consumes at least one serving (100 g) of kimchi daily, which is equivalent to 781 mg of sodium (Table 1). This amount corresponds to 52% of the adequate daily sodium intake (1500 mg) [52]. Apart from kimchi, daily Korean meal is also high in sodium, particularly because of the common practice of adding flavor enhancer such as monosodium glutamate to food and the high consumption of instant noodles [53]. High sodium intake has been scientifically proven to be associated with a higher risk of cardiovascular diseases [54]. Interestingly, the regular consumption of kimchi in the daily diet of Koreans was not associated with hypertension prevalence [55]. In contrast, high kimchi intake (210 g per day) was associated with a better lipid blood profile related to lower risk of cardiovascular diseases in humans [56]. Many scientists suggested that the overall healthy diet of Koreans comprising mainly vegetables that are rich in fiber and antioxidants but low in calories would compensate the high daily sodium intake.

## Eating kimchi is a communal activity

Korean food contains the philosophy of “*bibim*,” which refers to act of communicating with other people: sharing what is good and filling in what is lacking. Eating is viewed in as a communal activity that embodies such a philosophy. In Korea, the practice of common eating with family members or friends is recognized as a cultural hallmark. It is also an opportunity for sociability in addition to nourishment. People not only share a table, but also eat from the same dishes, including kimchi as a side dish. One would take some kimchi by their chopsticks from the middle of the table, then put it in their plate and eat it with some rice and other dishes. “*Hansotbap sikgu*” is a Korean idiom that means “we eat rice out of the same bowl.” In the past, Korean family members would mix a bowl of *bibimbap* (rice with vegetables, meat, and spicy soy sauce called *gojuchang*), the father would begin eating, and the other family members would eat out of the same bowl [57].

Kimjang culture is a perfect example of how kimchi unites people in a community and strengthens the social bonds among them. After preparing kimchi collectively, people usually continue their activity by dining together. Some seasoned cabbages are directly eaten as a salad without undergoing fermentation in large clay pots. This type of preparation is called *baechu geotjeori* kimchi, which is also known as fresh kimchi since it is consumed quickly after being prepared [7].

## Kimchi is a symbol of filial piety

Korean people share traditional values and normative family dynamics rooted in ancient Confucianism. Filial piety (also known as “*hyodo*” in Korean) is the central Confucian principle defined as a natural responsibility of an individual to serve and support their parents [58]. The spirit of filial piety can be observed in daily practices of Koreans that respect elders in social situations. It is also particularly evident in Korean food table manners: Older people are seated at or near the head of the dining table, guests are typically served from oldest to youngest, the eldest person at the table is the first to start eating, it is polite to use both hands when serving an elder, and no one leaves the table before the eldest finishes their food [59].

Filial piety is also engraved in the values of kimchi. Kimchi is generally appreciated for its crunchiness since uncooked vegetables are used as raw materials. However, older people often suffer from dental and digestive problems that make them unable to enjoy kimchi as much as younger people. To address such a limitation, special kimchi like *suk kkakdugi* and *suk nabadji* are carefully prepared for the elderly from pre-boiled vegetables such as radishes to soften its texture. In winter, a traditional gift consisting of a special white porcelain jar of *kamdongjotmu* kimchi and home-made rice wine (*makgeolli*) is offered to the elderly in the family as a sign of filial piety [36].

## Humanistic values of kimchi and kimjang

From the perspective of humanities, kimchi represents five distinctive values that root from the philosophy of life embraced by Korean people [39]. Such values are also incarnated in the spirit of kimjang. These values depict how kimchi can be a perfect representation of Korean people and their way of life.

First, its complementariness as a side dish to other staple foods. Rice is the main and staple food in Korean meal, while kimchi is a side dish. Therefore, kimchi is usually not eaten alone. With its distinguished salty and spicy taste combined with its crunchy texture, kimchi makes an excellent complementary dish for staple food. Kimchi supplements what is lacking in the staple food: it provides flavor to the bland taste of rice and additional nutrition (fiber, vitamins, and minerals) to the rice consisting mainly of carbohydrates. In this case, kimchi is a representative example of interdependence and together with other elements, kimchi composes a complete Korean meal rich in flavor and health benefits. Such a function reflects the communal philosophy of “being dedicated to helping others and benefiting the world,” as also observed in the spirit of the kimjang where people gather, work together, and help each other while preparing kimchi.

Second, kimchi is highly diverse. There are more than 200 varieties of classic kimchi that have been identified, and new kimchi variations are constantly emerging. The diversity of kimchi is mainly due to geographical factors, different ingredients used, different parameters applied for fermentation, different serving techniques, different flavor, etc. Indeed, Korean people respect and appreciate the diversity of kimchi as it also represents the diverse Korean culture and community. Kimjang is also a celebration of diversity, since it gathers people from different families and backgrounds for the same purpose, which is to make kimchi. During kimjang, different varieties of kimchi are also made, thus enhancing the essential signification of diversity of kimchi and kimjang culture.

Third, kimchi is a symbol harmony and independence. As previously stated, kimchi is an excellent example of diversity. With the globalization of kimchi, it has been incorporated in different local food cultures abroad. In Japan, it needs to become Japanese *kimuchi* to accompany Japanese sushi. In China, kimchi is harmonious to be consumed with Chinese dumplings. In America, kimchi replaces pickles in a hamburger. Such phenomena do not mean that kimchi loses its originality. Kimchi is independent and is still acknowledged as a Korean delicacy, but it can also be harmonious with any food, thus reflecting a strong adaptability toward a new culture. These humanistic values of kimchi can also be observed in the local kimjang culture that gathers different people, including newcomers from other regions. Korea is known as “*tongbangyeujuguk*,” the country of courteous people. Kimjang is a perfect occasion for newcomers to get acquainted with the locals in their new environment. Indeed, during the very event, these newcomers introduce the local kimchi recipes from their regions and they also learn the kimchi recipes originating from their new environment. Such a moment of exchange and sharing would reinforce the values of humanities among the people and enrich the food culture in the society.

The fourth value is kimchi’s long preservation process. Of numerous Korean foods, kimchi might be the only dish that require a long fermentation process at low temperature to ensure the availability of vegetables throughout the winter, during which no plant would be able to grow. Kimjang kimchi is kept in clay jars during the winter to ensure a long preservation period by slowing down the fermentation at low temperature. From a humanistic understanding, the long kimchi fermentation process would resemble the character building of human beings. Similar to the long fermentation of kimjang kimchi, it takes a relatively long period through different processes for a man to achieve a certain level of maturity. Throughout the slow fermentation process, kimjang kimchi develops a richer flavor and better organoleptic properties, just like human beings who gain knowledge and become wiser as they get older. The very moment of kimjang gathers people from different ages and generations. During the process of kimchi making, these people interact intensively with each other, thus allowing exchanges among them. People from the older generations would give advices and share their life lessons to those from the younger generations. Such a phenomenon would help the younger people in their adulthood. It is such a beautiful moment of sharing that takes place continuously in the history of humanity. One day, the young people taking advice will be the ones giving advice during the kimjang.

Fifth and lastly, kimchi brings harmony among its various ingredients and represents unity. In terms of taste, kimchi is a perfect example of harmony between sweetness, saltiness, sourness, and spiciness. It is harmonious as well in terms of ingredients and color. Every ingredient in kimchi is important and contributes to the whole flavor of kimchi, thus creating a unique kimchi flavor. If the taste of a specific ingredient is too strong, it would overpower other ingredients and disturb the kimchi’s identity. In the making of green pepper kimchi (*gochu sobagi*), the green pepper, which is used as the main ingredient, is soaked in brine solution or vinegar to remove its spicy taste so that its spiciness would not overpower the flavor of the other ingredients. Unity is the essential value of kimjang. During such a precious moment of togetherness, people from different backgrounds in the community gather without considering their economic or social status. They all work together in harmony for a common interest. In this case, kimchi acts as a symbol of unity, an agent that unites people.

## Conclusions

Kimchi and kimjang are Korea’s cultural assets rich in both historical and philosophical values that have been present for thousands of years with Korean people, from the ancient times to the modern days. During the time travel, kimchi and kimjang culture have changed their faces and adapted with the changes taking place in Korean society. Such a phenomenon has enabled kimchi and kimjang to survive until today. These elements are living proofs that food can unite people and people can find their identity and pride through food. The philosophical values of kimchi and kimjang culture explain how Koreans have respected the nature and traditions besides embracing the spirit of togetherness since the ancient times. These are the values on which the next generations are expected to rely. Kimchi and kimjang are the incarnation of the authentic Koreans’ spirit and way of life. The food reflects indeed the people, just like the classic popular quote of Jean Anthelme Brillat-Savarin: “you are what you eat.”

## Data Availability

The data and material used in this work are available upon request.
